# Beer, barley, livestock, milk: Who adopts agricultural innovations in rural Rajasthan?

**DOI:** 10.1016/j.wdp.2024.100643

**Published:** 2024-12

**Authors:** Dina Najjar, Bipasha Baruah

**Affiliations:** a Social, Economics and Policy Research Theme, Sustainable Intensification and Resilient Production Systems Program (SIRPS), International Center for Agricultural Research in the Dry Areas (ICARDA), Avenue Mohammed, Bearabi Alaoui, Agdal Hay Ryad, Instituts Maroc, Morocco; b Canada Research Chair in Global Women’s Issues, University of Western Ontario, Lawson Hall 3244, 1151 Richmond St, London, ON N6A 3K7, Canada

**Keywords:** Gender, Poverty alleviation, Agriculture, Rajasthan, India, South Asia, Intersectionality, Livestock

## Abstract

Research conducted in developing countries in the past 50 years generally suggests that most agricultural innovations (whether technological, social, or financial in nature) end up reinforcing existing socio-economic hierarchies based on gender and class. Most of these findings are drawn from the Green Revolution, which focused overwhelmingly on high-yielding varieties of rice, maize, and wheat, along with the introduction or expansion of irrigation and extension services and the use of fertilizers and pesticides. Less is known about how agricultural innovations involving other crops or livestock, especially if introduced in tandem, perform in alleviating poverty or reducing gender inequality. We conducted a study in three agricultural communities in rural Rajasthan, India to understand how the adoption of agricultural innovations for barley cultivation and livestock rearing are influenced by the gender, age, and class background of farmers, and whether such innovations can alleviate poverty and promote gender equality in rural settings. We found that although innovation adoption is influenced by gender, class and age (with gender exerting a stronger influence than class or age), poorer farmers and women can under certain circumstances benefit from agricultural innovations adopted initially by wealthier male farmers.

## Highlights

• Socio-economic hierarchies often shape the benefits of agricultural innovations.
• Research mainly focuses on Green Revolution crops like rice, maize, and wheat.
• Little is known about socio-economic impacts on other crops, livestock, and their interactions.
• Our Rajasthan study explored barley farming and livestock rearing.
• Poorer farmers and women can benefit if innovations are first adopted by wealthier men.

## 1 Introduction

Most social research on agriculture emphasize that better-resourced people, typically men and those from socioeconomically privileged groups, are able to reap more of the economic and social benefits from new agricultural technologies and innovations while less well-resourced farmers, including women and people from less privileged groups, either do not benefit equitably from, or, are disadvantaged further by new technologies and innovations (see, for example, Feldman and Biggs, 2012, Feldman and Welsh, 1995, Kerr, 2012, Krishna et al., 2020, Leach, 2015, Patel, 2013, Beuchelt, 2016). However, most of this research appears to be based upon innovations introduced during the Green Revolution in countries like India and Mexico, which focused mostly on high-yielding varieties (HYV) of rice, maize, and wheat, along with the expansion of irrigation and extension services, and the use of fertilizers and pesticides. Less is known about how innovations involving other crops or livestock, especially when introduced in tandem, perform in alleviating poverty or reducing gender inequality.

We conducted a study in three agricultural communities in rural Rajasthan, India, to gain a more nuanced understanding of how the adoption of agricultural innovations for barley cultivation and livestock rearing are influenced by the gender, age, and class background of farmers, and whether such innovations can alleviate poverty and promote gender equality in rural settings. We used data from focus groups with women and men from different economic backgrounds and generations, who may or may not have adopted the innovations for barley cultivation and livestock rearing, as well as in-depth semi-structured interviews with men and women farmers who had adopted the innovations. We found that the capacity to adopt barley and livestock innovations was influenced quite strongly by the gender, age, and socioeconomic background of the farmer. To improve the ability of different groups of farmers to adopt agricultural innovations and to benefit optimally from them, scientists, governments, donors, the private sector and other actors in the agricultural sector should pay more nuanced attention to these intersecting identities.

## 2 Gender, class, and adoption of agricultural innovations

Much research has been conducted since the 1970 s to understand the longer-term effects and outcomes of the Green Revolution in countries like India and Mexico (Krishna et al., 2020, Moseley et al., 2015, Patel et al., 2015, Vercillo et al., 2015). The existing literature on the socioeconomic outcomes of the Green Revolution and on other agricultural innovations in more recent years tend to concur that small-scale marginal peasant farmers and women farmers did not receive many of the benefits of agricultural innovation during the Green Revolution and were perhaps marginalized even further because of it (Krishna et al., 2020, Moseley et al., 2015, Patel et al., 2015, Vercillo et al., 2015). Since the Green Revolution focused primarily on providing a technological package of HYV seeds, fertilizers, and pesticides along with extension services, most of which farmers had to fund through cash flow or credit, wealthier farmers with larger farms benefited more than poorer farmers (Clawson and Hoy, 1979, Sonnenfeld, 1992). HYV wheat, for example, needed at least 15 acres of cultivable land to be viable under ideal conditions, thereby rendering small-scale, marginal, and women farmers mostly unable to benefit from them (Freebairn, 1995). The Indian government did make some effort to render the package accessible to small farmers but due at least in part to entrenched class, caste and gender inequalities in India, the benefits of the Green Revolution were reaped disproportionately by wealthier upper-caste farmers (Dhanagare, 1987).

This tendency of most agricultural investment policies associated with the Green Revolution to intentionally or unintentionally “bet on the strong” is also at least partially what led to the decline of production of other crops that are important from a nutritional perspective, such as millets, sorghum, and lentils (Lerner, 2018). More than 86 percent of farmers in India cultivate less than two hectares of land (Saini and Chowdhury, 2023). Poorer farmers tend to be more risk averse and are also more likely than wealthier farmers to live in a wider range of agro-environmental zones. Poorer farmers tend to prefer planting food crops, often simply because they cannot access the type of land, inputs and economies of scale required to benefit from growing cash crops. Small farmers tend to plant a variety of food crops both to optimize household food security and to manage risks such as drought, flooding, and high winds (Becerril and Abdulai, 2010, Brush et al., 1988, Smale et al., 2001).

Although it is widely accepted that the Green Revolution increased total food production and supply at the national level in countries like India and Mexico, whether it improved nutrition at the household level is debatable. The existing data about national food supply and access enabled by the Green Revolution is overwhelmingly positive but may be misleading since it also does not consider the intra-household distribution of food (Negin et al., 2009, Pinstrup-Andersen and Hazell, 1985). Since adult men tend to consume more food, women and children may suffer nutritional deficiencies even when the household is deemed to have sufficient food (see, for example, Harris, 1990, Harris-White, 1997). Nutritional adequacy is also dependent on access to protein and micro-nutrients derived from a variety of foods; not simple calorie intake derived from carbohydrate-rich cereals. In India, for example, increased wheat production may have happened at the expense of protein-rich beans, millets, and lentils (Harwood, 2012). Micronutrients found in foods such as fish in rice paddies, and wild leafy vegetables also became less available as wheat took up more acreage (ibid).

Other distributional effects of the Green Revolution became clearer when tensions emerged between wealthier land-owning farmers and landless laborers who were not seeing commensurate benefits, for example via increased agricultural wages, from the new agricultural technologies and services (Frankel [1971] 2015). The success of the Green Revolution also changed the political landscape in India, giving wealthier land-owning (mostly male) farmers a stronger political voice that ensured the continued protection of the larger-scale agricultural sector via access to credit and subsidies, for example, often at the expense of the poorer marginal farmers (Das, 1999, Cleaver, 1972, Kaviraj, 1988).

The existing social research literature published during and after the Green Revolution often presents the roles that are played by (or that could potentially be played by) the state and the private sector in introducing and diffusing agricultural innovations as somewhat conflictual and mutually exclusive of one another (see, for example, Kilby, 2019). The general tendency in this literature is to assume that the state must solely or overwhelmingly be responsible for leading efforts to promote social equity in agriculture. While some authors have acknowledged that other actors such as donors, international and local development banks and financial institutions, NGOs and civil society actors can play important complementary roles in alleviating poverty or promoting gender equality (Baruah, 2007, Baruah, 2021), the private sector is rarely envisioned or expected to facilitate anything other than the generation of profits for shareholders, especially in the agriculture sector. The general agreement within this literature appears to be that the entry of the private sector into any aspect of agriculture can only lead to the exacerbation of existing social inequalities and hierarchies (Kilby, 2019). The possibility that “corporate interests” in agriculture might be reconcilable in some contexts and under specific circumstances with poverty alleviation and gender equality is rarely, if ever, explored in the existing literature on gender, class, and adoption of agricultural innovations.

## 3 Background and Rationale for study

Along with Uttar Pradesh, Rajasthan is one of the largest barley-producing states in India, accounting for more than 50 percent of total barley harvested and nearly 50 percent of total barley acreage in India (ICAR-IIWBR, 2017). Rajasthan is mostly an arid desert state. The Thar Desert, also known as the Great Indian Desert, is the world's 17th largest desert and covers a significant part of western Rajasthan. The average annual rainfall in east and west Rajasthan is about 64.9 cm and 32.7 cm respectively. The southern and southeastern districts of the state receive about 50 cm and 43 cm respectively. Wheat is grown in regions of the state where irrigation is available, but barley is also often also grown in these regions because although barley has a lower market price than wheat, it produces a higher yield than wheat in drought conditions and can tolerate salinity better than wheat (Newton et al., 2011). Barley can also be grown in lighter and looser soils than wheat can withstand, another reason why barley is grown in Rajasthan. Livestock farming has also historically been important in dry and desert regions, especially as a protective measure against crop failure (Fafchamps et al., 1998, Hänke and Barkmann, 2017). Although barley is generally grown as a food crop in Rajasthan, some communities prefer growing forage barley because unlike food barley, forage barley can be harvested twice per season and used to sustain livestock (Newton et al., 2011, ICRISAT, 2012).

In recent years, barley has also gained prominence in Rajasthan as a cash crop due to the use of barley malt in the production of beer (Pfitzer and Krishnaswamy, 2007, Singh, 2013). Beer consumption has grown dramatically in India in recent years owing largely to a preference among younger urban Indians for beer over other types of alcoholic beverages. Since more than 65 percent of India’s 1.4 billion population is below the age of 35, the demand for beer (and therefore barley malt) is expected to remain strong for the foreseeable future. Several private sector international manufacturers such as SABMiller and Pepsico recently entered the Indian beer market, which is estimated to be growing at 15 to 18 percent per year (Times of India, 2009, Singh, 2013). Because the availability in India of barley varieties suitable for malting was low and the supply precarious, these manufacturers had initially begun operations in India by importing malt. More recently, they have started entering into agreements for barley cultivation with farmers as imports proved to be very expensive. Approximately 10,000 farmers in Rajasthan are now part of SABMiller’s barley supply chain and PepsiCo has signed a memorandum of understanding with more than 1,200 farmers in Rajasthan to produce high-yielding malt barley for the United Breweries Group (SABMiller, 2010, Singh, 2013, Times of India, 2009).

The Indian Institute of Wheat and Barley Research (IIWBR) collaborates with 8 other partner institutions to conduct research on barley in India through the All India Coordinated Wheat & Barley Improvement Project. This “Barley Network” is promoting the cultivation of barley to conserve water resources and to meet the growing demands of brewing companies, such as the United Breweries Group and SABMiller that breed and popularize varieties of barley which are high yielding and have better malting qualities (Times of India, 2009). These efforts are concentrated both in the very dry areas of Rajasthan that generally grow barley and in partially irrigated areas that also grow wheat (Newton et al., 2011). According to the Barley Network, these efforts are modestly reversing some of the effects of the Green Revolution, which had dramatically increased wheat production and depleted groundwater resources (Kerr, 2012). One of the barley breeders we interviewed for this study emphasized that because so much acreage in India was dedicated to wheat during the Green Revolution, barley eventually became a “forgotten crop” and improved barley seeds became more difficult to develop and distribute. As opposed to wheat, which benefits from a government-organized procurement system with a Minimum Support Price (MSP), barley farmers must figure out a way to store their harvest and transport it at their own cost and sell it when prices are optimal in the open market since there is no organized government procurement for barley in India (Singh, 2013). The volatility of barley prices, especially when compared to wheat, was also a disincentive for farmers to grow the crop (ibid.).

As mentioned earlier, recent interest in barley, especially from the private sector, can be attributed to a growing consumption of beer in India and South Asia, and subsequent demand for barley grain for malting purposes. Barley breeding efforts are now concentrating on malt varieties that have an elevated starch and enzyme content suitable for the brewing process. Nonetheless, private brewing companies also continue to depend on feed grade barley, which is higher in protein and β-glucan contents, due to the limited availability of malt varieties. Feeding barley grain to livestock leads to an increase in milk and meat yield while feeding barley straw to livestock leads to increased fat content in milk, which is a desirable trait (Singh, 2013). In recent efforts to popularize or reintroduce barley in India, new breeds of buffalo, cows and goats are introduced simultaneously alongside improved barley varieties and related agronomic practices to optimize outcomes for farmers of both barley and livestock cultivation. Improved livestock breeds tend to produce more milk, although some may also be more susceptible to disease.

Given such interdependencies, agricultural innovation in barley and livestock rearing tend to influence one another. Recent studies that looked closely at the interplay between agriculture and animal husbandry found that social identities based on gender, age, and social class influence whether farmers can adopt and benefit from innovations in these fields (see, for example, findings from rural Pakistan in Drucza and Peveri, 2018). Building upon such previous research, we looked at how gender, age and social class might influence the ability to adopt and benefit from innovations in barley and livestock cultivation in three rural communities in Rajasthan.

## 4 Background information about study areas

**Fig. 1:**
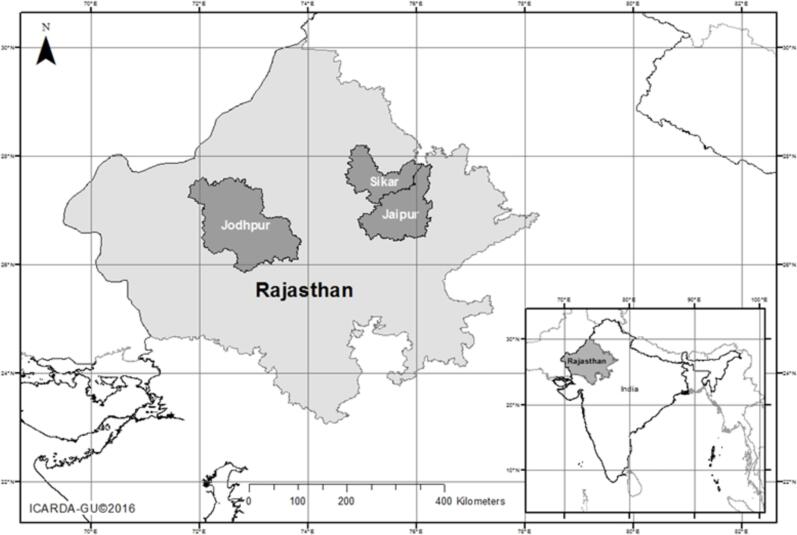
Study Areas in Rajasthan ().

Source: ICARDA Geoinformatics Unit 2017

The three communities in Rajasthan included in this study were chosen purposely based on differences in access to agricultural markets and services as well as existing gender norms. We use acronyms for the names of communities and pseudonyms for our informants in accordance with the stipulations of the research funding from the Consultative Group for International Agricultural Research (CGIAR) for this specific project entitled Gennovate. While EB (in Jaipur district) and MU (in Sikar) are better connected to markets, MA (in Jodhpur) is more isolated, with poorer access to agricultural services, including markets (Fig. 1). Women’s participation in agriculture, markets and community institutions also differed significantly in the three regions. Broadly speaking, MA appeared to have more restrictive social norms for women than EB and MU. For example, *purdah* or female seclusion was practiced only in MA, and women almost never inherited land in MA but occasionally did so in EB and MU. It would be unwise to speculate why MA is more socially conservative than the other two communities. Village leaders in MA and staff from agricultural research organizations suggest that being farther away from major urban centres and services may at least partially explain why traditional social norms were more pronounced and persistent in MA. We summarize socio-economic characteristics and norms in the 3 communities in Table 1.

**Table 1:** Socioeconomic characteristics and norms in the 3 communities.

**Characteristic**	**EB**	**MU**	**MA**
Population	123,000	18,570	80,000
Average farm size	1 ha	1 ha	5 ha
Irrigation	Sprinkler, drip, and rainfed	Sprinkler, drip, and rainfed	Sprinkler and rainfed (irrigation availability is lower than the other two communities and groundwater is saline)
Ethnic/caste groups	Rajput, Jat,Yadav*, Brahmin*, Mali, Meena	Saini*, Rajput*, Raiger, Brahmin*, Yadav*, Kumawat	Gurjar, Jat, Bishnoi*, Brahmin*, Meghwal, Rajput*
Families	Mostly extended, few nuclear	Mostly extended, few nuclear	Almost all extended
Women who work for pay now and ten years ago	30 % and 10 %	17 % and 5 %	30 % and 5 %
Women and market access	Regularly sell in local markets	Occasionally sell in local markets	Rarely sell in local market
Women and farming	Women manage farms and women farm land owned by husbands	Women manage farms and women farm land owned by husbands or fathers in law	Women do not manage farms or own land
Inheritance	Men inherit land; women inherit only if there were no men inheritors.	Most land is inherited by men; a few women inherit land, but most get livestock (cows, buffalo and goats) from parents.	Women get money and livestock from parents, almost never inherit land.
Access to Schooling	Upper secondary school is available. Almost all girls and boys attend primary school. Almost all girls attend secondary school; only 75 % of boys attend.	Upper secondary school is available. Almost all girls and boys attend primary school while 75 % of boys and girls attend secondary school.	Lower secondary school is available. Almost all boys and girls attend primary school; 75 % of boys and 50 % of girls attend secondary school.
Temporary male outmigration	One fourth of households in the community	One fourth of households in the community	One fourth of households in the community
Temporary female outmigration	Almost none	Almost none	One fourth of households in the community (women migrate with husbands)
Share of Women Headed Households	5 %, due to widowhood and illness of men	10 %, due to widowhood, migration and illness of husbands	8 %, due to widowhood, migration and illness of husbands
Gender Wage Gap	28 % in agriculture, 40 % in non-agricultural work	17 % in agriculture, 50 % in non-agricultural work	25 % in agriculture, 40 % in non-agricultural work
Gender of local officials in past 10 years	Women have been elected to lead; 33 % of council members are women.	Women have been elected to lead; 33 % of council members are women.	Women are never elected to leader; 33 % of council members are women

*Castes that are better-off economically and more politically powerful.

The three communities also have different histories and experiences with barley cultivation. While the MU and EB communities have been growing barley for fodder for several decades, MA has been growing barley for fewer than ten years. Barley farming was only reintroduced in MA in 2013–2014 by the International Centre for Agricultural Research in Dry Areas (ICARDA) with support from the Central Arid Zone Research Institute (CAZRI) in Jodhpur. Ten farmers (all men) were selected in MA by ICARDA and CAZRI based on availability of land and their willingness to participate in growing and demonstrating new barley feed varieties (RD 2786 and RD 2660), alongside related improved agronomic practices, early planting, fertilization, and irrigation practices. Seven farmers were given one variety of barley and three were given both. A scientist and an extension agent made multiple visits to the hosting farmers and invited other farmers to attend to demonstrate the benefits of using improved barley varieties along with related agronomic practices. Two trials were run simultaneously on each farmer’s land, one using local varieties and another using the improved varieties along with improved agronomic practices such as early sowing, proper fertilization dosages and timing, irrigation dosages and timing, and disease identification and control. On average, the improved varieties produced 15 percent more grain and 10 percent more straw than the local varieties. These results were particularly impressive in MA since it is the driest of the three communities, and one that encounters a severe drought every four to five years.

In MU, in the district of Sikar, five male farmers hosted what were called “Front Line Demonstrations” in 2014 for popularization of barley variety BH 902, which was adopted under the auspices of the Barley Network in India as a feed barley cultivar for irrigated conditions. A team of experts advised farmers in MU on agronomic practices. In this village, attention was also paid to *Bhual* goats, which have been introduced to the village over the past ten years. This breed produces 4–5 kids every 6 months instead of the average of 2 kids produced by the local breed. *Bhual* goats also produce 2–4 times more milk than the local breed and are more resistant to diseases. There is an increased demand for goat milk in the region, in part because of a popular but false belief that goat milk has medicinal value in treating dengue fever, tuberculosis and diabetes.

In EB in the district of Jaipur, SABMiller has been supporting contract farming with malting barley cultivars since 2010, as part of a program called *“Saanjhi Unnati”* (Progress through Partnership) in four states in India (SABMiller, 2010). The project provides its own extension support to the farmers via over 60 extension agents spread over the participating states. Most of the extension agents are men. Some of the participants in the program are women, although due to cultural norms, the person in whose name the project is registered is almost always a man. The program provides farmers with barley seeds and other agricultural inputs, the cost of which gets deducted from the harvest sold to SABMiller’s barley supply chain. To ensure the timely availability of good quality barley grain as raw material for the malting industry, SABMiller buys the barley harvest from contracted farmers at a higher price than its market value, which is often volatile (Singh, 2013). Since the Indian government had already hosted barley demonstrations in EB through farmer field days and schools prior to the launch of the *Saanjhi Unnati* program, many of the local farmers were already familiar with the benefits of adopting improved barley varieties and improved agronomic practices.

## 5 Methods

Our primary research for this study included conducting focus groups with community members and interviews with community leaders, early adopters of agricultural innovations, and barley breeders. To compile the table of socioeconomic characteristics of the 3 communities (Table 1), we synthesized available data from local government councils and interviewed community leaders.

Focus group discussions and interviews were conducted between October and November 2019 in MU (Sikar), EB (Jaipur) and MA (Jodhpur). Follow-up data collection aimed primarily at clarifying findings and filling data gaps was conducted via phone interviews ,because COVID made it impossible to return to Rajasthan to do more fieldwork, between March and July 2021. In total, 224 community members from EB, MU and MA participated in this study: 180 individuals in focus groups, 12 interviews with early adopters, and another 12 with community leaders.

The focus groups were comprised of 10 unrelated participants, at least 6 of whom had to be involved in agriculture. Groups were formed based on gender, socioeconomic status, and age to minimize power differences and offer safe spaces for people to have freer discussions. Socioeconomic status was determined based on landownership as follows: middle-class (2.5 or more hectares of land) and poor (2.5 or less hectares of land or landless). Youth were defined as those between the ages of 16 and 24. In addition to the age criterion, we ensured that more than half of the youth participants were involved in farming, that they were not from the same family, and that they represented different life stages (married, unmarried, graduate, student). The men and women respondents in our interviews and focus groups were from different households. Based on these parameters, we conducted a total of 6 focus group discussions in each of the 3 communities: 1 with middle-class men, 1 with middle-class women, 1 with poor men, 1 with poor women, 1 with young men, 1 with young women.

The study sought to understand the differences between men’s and women’s capacities to adopt and benefit from barley and livestock innovations. In each focus group, participants were asked to describe and discuss who is considered a farmer in their community; whether women can access credit, extension services and other inputs into farming at par with men; which agricultural innovations were preferred by men and women, and why. They were not provided with a list to choose preferred innovations from. We simply recorded the innovations that were identified. Participants were also asked to reflect on changes, if any, in local gender norms (education, employment, access to markets, inheritance practices, community leadership) over the past ten years, and to describe changes in their families and communities due to shifts in gender norms.

We conducted 4 interviews in each community with people deemed to be early adopters of the innovations being studied; two were men and two were women. We asked innovators why they adopted the barley and/or livestock innovations, if they had reaped benefits because of adoption, and what those benefits were. In each community, two male and two female community leaders (such as government official, politician, lead farmer, or schoolteacher) were also interviewed to gain a deeper understanding of socio-economic characteristics and community-level factors that affect women’s and men’s roles and activities in each community. These included men’s and women’s roles in agriculture, local governance, participation in markets, outmigration from the community, types of families present in the community, access to education, and norms for inheritance.

Finally, 3 interviews were conducted with barley breeders employed by the International Center for Agricultural Research in the Dry Areas (ICARDA), Rajasthan Agricultural Research Institute, Jaipur (RARI), and SABMiller (these breeders are also members of the Barley Network of India) to better understand innovations in barley cultivation, the existence or lack thereof of supportive government policies, and the actual and potential outcomes of barley cultivation in the three study sites. Two men and two women with graduate degrees from universities in Rajasthan conducted the interviews and focus groups, with one asking the questions and another taking notes. They were trained over a ten-day period on the methodology and participant selection criteria by the first author of this paper.

We used inductive issue identification and explanation building from the focus group and interview data to generate findings from this study. Inductive analysis entails allowing the issues to emerge from the data. We conducted a knowledge synthesis of existing scholarly and practitioner literature on how adoption of agricultural innovation is influenced by gender, age and class prior to carrying out the fieldwork for this study, but we did not approach the focus groups and interviews with preconceived themes or issues we expected to encounter based on the literature review. We triangulated findings from the primary (focus group and interview) data and secondary (literature review) data to generate external validity for our findings.

## 6 Findings and discussion

We are presenting the findings in this section as themes based on the questions we asked in the focus groups. Of course, the issues discussed under each theme are related to one another (for example, references to social norms appear throughout although we also devote a brief separate section to discussing norm changes). Therefore, we ask that the reader approach the themes with their interrelatedness in mind.

### 6.1 Who is considered a farmer?

In all three communities, only men were identified as farmers in the focus groups and interviews. We found that although women in all three communities cultivated and managed farms and sharecropped land, often due to male outmigration or illness and widowhood, women almost always described themselves as playing supportive roles to men in agriculture. Women did not see themselves as farmers and regardless of the extent of their knowledge of and contributions to agriculture, both women and men deemed “helping men” to be the most appropriate role for women to play in farming. By contrast, men were expected to take on leadership roles in farming, which included not just being knowledgeable about farming practices and market prices and dynamics but also being able to take risks by adopting new technologies or farming practices.

This finding is not unique to our study or to rural Rajasthan. The fact that women farmers are perceived (and often perceive themselves) as helpers to men farmers rather than as farmers has also been documented extensively in the literature on gender and agriculture in South Asia, Middle East and North Africa (MENA), and Sub-Saharan Africa (see, for example, Agarwal, 1994, Bigler et al., 2017, Dewan, 2016, Najjar et al., 2020). Therefore, it follows that the existing literature emphasizes the importance of enabling women to gain both legal and social recognition (including self-recognition) as farmers (see, for example, Agarwal, 2003, Baruah & Najjar, 2022, Kilby, 2019). Although there is no consensus in the literature on how to accomplish this, there are suggestions that different institutions such as governments, donor agencies, banks, domestic and international agricultural institutions, and the media can play a role in enabling this by reforming male-biased inheritance and property ownerships laws for greater gender equity (Agarwal, 1994, CABI., 2020), enabling cultivators of land to gain access to agricultural inputs and services at par with land owners (Kilby, 2019), and by supporting efforts to raise public awareness about women as famers, their contributions to national economies, and to society at large (Baruah & Najjar, 2022, Najjar et al., 2018).

### 6.2 Access to credit, training, and agricultural extension services

We found that the lack of recognition of women as farmers also influenced access to credit, information and training about farming practices, and agricultural extension services in all three communities. Men generally had much higher levels of entitlement and better access to such services than women from the same households. These issues have been well known for decades. For example, Pearse ([1980] 2015) documented the different risk profiles of larger and smaller farms and women farmers, and their inability (particularly for women) to access credit for their inputs. Since then, other researchers have written extensively about these associated challenges and outcomes of the lack of legal and social recognition of women as farmers and their inability to access land ownership at par with the men in their families and communities (Doss et al., 2015, Kilby, 2019, Pearse, 2015). We found that although women in the study communities often understood the value of learning about irrigation or adopting new agricultural practices, men did not support or acknowledge women in these roles and rather focused on more traditional roles related to domestic responsibilities. Thus, even when (as in EB and MU) women were not prevented by *purdah* (female seclusion) norms from attending training or meeting with (male) extension agents, they rarely did so. We found that women from better-off families in all 3 communities were more likely to adhere to social norms of seclusion*,* often simply because they could afford not to work outside the home, and they were even less likely than poorer women to attend meetings and seminars organized by men extension agents. Women in all three communities emphasized that having access to women extension agents could improve their access to farming information. They identified lack of access to extension workers as the main reason why men usually adopted innovations before women did. While acknowledging a preference for women extension agents, women in EB and MU emphasized that all extension agents should be encouraged to reach out to and work with both men and women farmers. For this to happen, of course it was particularly important for extension agents to think of women as farmers rather than as helpers and to reach out them proactively with extension services and training. Other researchers have also emphasized the value both of training more women as extension agents while simultaneously encouraging extension agents of all genders to reach out to women farmers (Buehren et al., 2017, Manfre et al., 2013, Ragetlie et al., 2022, Twyman et al., 2015).

The lack of or poor access to extension services meant that women in all three communities typically accessed information about new agricultural innovations indirectly through their husbands or other men in the community. In some cases, women learned from other women, who were usually the wives of male early adopters of agricultural innovations. The only instance in which women had direct access to information about agriculture was in EB, where some women were part of the local government council and were therefore invited to meetings. In turn, they encouraged other women to attend the meetings and to participate more actively in the barley adoption program. Meetings in EB were held in the dairy centre or the panchayat (local government council) building, locations that were familiar to everyone in the community, which also encouraged higher levels of attendance by women. Other researchers have emphasized the importance of paying attention to the particularities of women’s needs, challenges, and priorities in specific cultural contexts in developing extension services and optimizing farmer participation in training. For example, Kilby (2019) notes that training should be tailored to times and venues that suit women, and that these may be different from those that suit men.

We found that men in all three communities were much more likely to access credit via formal loans from banks, state-run and private sector agricultural organizations, and farmers’ cooperatives. Women had much weaker access to credit from formal institutions, and they were much more likely to access credit via informal means such as loans from family members, self-help groups or moneylenders. Consequently, women were much more vulnerable to the unscrupulous or exploitative practices of informal moneylenders. Strengthening women’s access to formal financial institutions could help alleviate their dependence on informal sources of credit. Researchers working in rural agricultural contexts in MENA and Sub-Saharan Africa have also made the same observation (see, for example, Najjar et al., 2019, Baada et al., 2019, Pearse, 2015).

We found that women may face more challenges accessing formal credit because land, which women rarely own, is often used as collateral by formal credit-granting institutions. Women’s marginalization in land ownership has negative implications for women and household prosperity more broadly, including adoption of new barley varieties and improved livestock breeds, especially if the women were the heads of households, as was estimated for between 5 and 10 percent of households in the three villages in Rajasthan, largely because of male outmigration and widowhood. Reforming male-biased inheritance practices and/or encouraging financial institutions to waive collateral requirements (especially for women borrowers) to access financing are both necessary to reduce the gender gap in access to financing.

### 6.3 Agricultural innovations deemed most beneficial

Our research revealed clear gender-specific preferences for innovations. All respondents (men and women) identified innovations related to farm machinery, pesticides, and irrigation as being the most useful for men. Similarly, both women and men identified innovations associated with dairy processing, livestock, and feed preparation as being most helpful for women. These preferences are understandable since sociocultural norms ascribe women with responsibility for food provision and preparation at the household level, and men with the broader economic survival and security of the household. The innovations listed above were also preferred by men and women across community, class and age categories. In other words, gendered social norms and expectations may transcend class and age, which is why gender appeared to play a much bigger role in innovation preferences than class or age. These findings resonate with those of Kilby (2016, 67) who emphasizes that “the farming household is complex, and women and men have different management roles and often grow different crops, and as a result have different needs in terms of agricultural technology and support.” Others have noted that because households are complex systems, gender must also be understood “in the context of age, caste, and livelihoods” (Fisher and Carr, 2015, 83).

We also asked participants to evaluate the usefulness of the recent innovations in barley and livestock (new goat, buffalo, and cow breeds, for example). The new barley varieties were evaluated most positively by men from poorer backgrounds for several reasons: the yields were almost twice as high as the previous variety; barley requires less water than other crops; and farmers have a guaranteed market via the beer malting industry, and they can sell the grain at a higher price than was previously possible. Other researchers who have observed that the large-scale adoption by farmers of maize, rice and wheat during the Green Revolution was at least in part influenced by the role played by governments in guaranteeing prices and profits to farmers and developing supportive financial services such as credit and loans and physical infrastructure such as roads, rail, and collection centres in rural locations (Harwood, 2012, Kilby, 2019). Our findings from Rajasthan confirm the value of such supporting services and policies in ensuring that agricultural innovations gain optimal traction among farmers. Our findings do differ from those of other researchers in that we found that supportive services, policies, and infrastructure can also be designed, delivered, or optimized through the efforts of other actors in agriculture, notably the private sector and agricultural research institutions.

Our findings revealed that both livestock and barley innovations were often adopted first by wealthier male farmers and eventually adopted by less well-off farmers. This finding from rural Rajasthan complicates the general assumption in the literature (see, for example, Kerr, 2012; Patel, 2013) that the adoption of agricultural innovations often results in a zero sum-game of wealthier farmers benefiting at the detriment of poorer farmers, or men benefiting from innovations at the expense of women. Other researchers (Lerner, 2018, Perkins, 1990) have pointed out that the targeting of agricultural innovations to wealthier farmers and larger farms often makes sense for creating larger-scale production outcomes since such farmers have higher tolerance for risk and can therefore make more effective use of new inputs. These researchers simultaneously emphasize that the adoption of agricultural innovations by wealthier farmers leads to a “scale bias” (Pingali, 2012) and that the benefits of the innovations do not usually trickle down to women or poorer farmers, especially without ongoing government support (Kilby, 2019, Wellhausen, 1976). Contrary to such findings, in rural Rajasthan we found that the successes enjoyed by wealthier farmers in adopting the new varieties of barley and new breeds of goats, cows or buffalo appeared to have a strong demonstration effect upon others irrespective of gender, class, and age. The ongoing support from national and state governments as well as private sector institutions such as United Breweries Group, SABMiller and Pepsico, and agricultural research institutions such as ICARDA, CAZRI and IIWBR may have played an important role in enabling a wider array of farmers to benefit from innovations initially mostly adopted by wealthier male farmers on larger farms. These findings from our study in Rajasthan also contradict the conclusion other researchers have arrived at that poverty alleviation and gender equality in agriculture are best driven by the state, and that the involvement of the private sector can only entrench and amplify existing social hierarchies and inequities. We found that the state and private sector can under certain circumstances play complementary mutually supportive roles in promoting social equity in the adoption of agricultural innovations.

Women across class categories identified new breeds of livestock as the most useful innovation, due largely to higher milk and meat yields. Higher milk yields were identified as particularly important by women because they tend to exercise a higher level of control over income from milk sales than from other products. That women tend to have higher level of control over income generated from milk and milk products has been observed in other studies (Najjar et al., 2017). The introduction of new barley varieties, often due to demand for barley grain for malting, although targeted almost exclusively at men farmers, has also had strong positive effects upon local food security, household nutrition, and women’s income because once the barley grain used for malting beer has been sold off, the barley stalks are used as livestock feed. Thus, contrary to other researchers (see, for example, Frankel [1971] 2015) in our study in rural Rajasthan we found that even the landless poor benefitted through contract farming on wealthier farmers’ land from the introduction of improved varieties of barley. These findings reveal that the nature of the innovation and its interaction with the social, cultural, and geographic context in which it is introduced determines whether and how its benefits accrue to different groups of people.

Our findings from Rajasthan also complicate the neat distinction that exists in social science research on agriculture between “food crops” and “cash crops” and the argument that growing cash crops compromises the food security of poor rural communities. As the barley example demonstrates, food security and cash cropping do not have to be mutually exclusive, especially since the same plant can produce a cash crop as well as a food or fodder crop. Thus, while most women respondents in our study evaluated the new barley varieties very positively for their increased yields, resistance to water scarcity, and the extra household income earned by men from selling barley grain for malting beer, they also valued the increased fodder supply for fattening livestock and increasing milk supply. The potential value and use of cash crop “residue” is rarely accounted for in agricultural research. Local innovation for combining cash cropping with food security is also rarely researched and recorded. These findings from rural Rajasthan also point to the value of studying livestock and crop agriculture in an integrated manner rather than as discrete parts of farming systems.

### 6.4 Changes in gender norms

Respondents in our study attributed higher levels of education for girls and women and access to (limited) income earning opportunities, such as milk and dairy product sales as well as formal jobs such as school teaching and tutoring, to modest changes in women’s and girls’ agency and freedom over the past decade. Focus group participants of both genders in all 3 communities and community leaders emphasized that women had more decision-making power over household finances than they did ten years ago, mostly because of greater economic contribution to the household. We found that age and life stage were significant factors in influencing women’s household decision-making power. Older married and widowed women typically enjoyed more agency and freedom than younger married or single women. This has been observed in other rural agricultural settings (see, for example, Kabeer, 2018, Najjar et al., 2020). We did not, however, find evidence of deeper transformative changes in gender norms represented by, for example, women’s land ownership or political leadership in our study in rural Rajasthan. Women managed farms in both EB and MU but only inherited land if they had no brothers. In MA women neither managed farms nor inherited land. Even if they did not have brothers, in MA land was titled in the names of other men relatives.

In 1992, the 73rd Amendment to the Constitution of India, enabled the reservation of one-third of seats on local governance institutions for women (Brule, 2020). Although it has been criticized for being tokenistic, especially in the absence of other measures to promote gender equality, this intervention was intended to strengthen women’s ability to participate in local-level politics and to foster rural development. Our findings from rural Rajasthan suggest that women are largely excluded from decision-making related to local institutions and common resources in all three communities included in our study. Although women constitute 33 percent of local council members in all 3 communities, community leaders noted that their membership was largely nominal; most women were either not active in their roles or served as proxies for husbands and other relatives. In the past decade, several women have been elected to leadership positions in the local government council in EB and MU, but there was no record of women being elected leaders in MA. Encouraging more women to participate in local governance institutions could lead to longer-term changes that normalize women’s presence and participation in the public sphere.

## 7 Conclusion

Social science research on agricultural development conducted in the past five decades (including research conducted during and after the Green Revolution) tend to conclude that most agricultural innovations (technological, social and financial) end up reinforcing existing socio-economic hierarchies based on class and gender (Krishna et al., 2020, Beuchelt, 2016, Leach, 2015, Kilby, 2019). We arrived at somewhat different conclusions in our study in rural Rajasthan. First, we found that although innovation preference is influenced by gender, class and age (with gender exerting a stronger influence than class or age), the adoption of agricultural innovations does not always result in a zero sum-game of wealthier farmers benefiting to the detriment of poorer farmers, or men benefiting at the expense of women. Instead, we found that the successes enjoyed by wealthier farmers in adopting agricultural innovations can under some circumstances also motivate poorer famers and women farmers to adopt the same innovations, especially with sufficient inputs and policy support from governments, private sector organizations, and agricultural research institutions.

Second, our findings from Rajasthan complicate the neat distinction that exists in the literature between food crops and cash crops, and the assumption that growing cash crops compromises the food security of poor rural communities. Based on the interaction between new barley varieties and new breeds of goats, cows and buffalo in rural Rajasthan, we found that the two can be mutually inclusive, especially because the same plant produces a cash and a fodder crop. In other words, food security and cash cropping can be accomplished simultaneously under certain circumstances in rural communities. This finding highlights the need for more research on the value and use of cash crop “residue,” and the importance of understanding local innovation for combining cash cropping with food security. More broadly, it highlights the importance of studying livestock and crop agriculture concurrently rather than as disconnected parts of agricultural systems.

Finally, findings from our study in Rajasthan complicate the assertion in the existing literature that poverty alleviation and gender equality in agriculture are best driven by the state, and that the involvement of the private sector can only entrench and amplify existing social hierarchies and inequities. We found that ongoing support from national and state governments, domestic and multinational private sector institutions, and domestic and international agricultural research institutions may also enable a wider array of farmers to benefit from agricultural innovations that may have initially been adopted only by wealthier farmers on larger farms. Our findings from the tandem introduction of new varieties of barley and livestock in rural Rajasthan broadly suggest that the nature of the innovation and its interaction with the social, cultural, and geographic context in which it is introduced determines whether and how its benefits accrue to different groups of people.

## CRediT authorship contribution statement

**Dina najjar:** Conceptualization, Funding acquisition, Methodology, Project administration, Supervision, Writing – original draft, Writing – review & editing. **Bipasha Baruah:** Conceptualization, Investigation, Writing – original draft, Writing – review & editing.

## Declaration of competing interest

The authors declare that they have no known competing financial interests or personal relationships that could have appeared to influence the work reported in this paper.

## References

[b0005] Agarwal B. (1994).

[b0010] Agarwal B. (2003). Gender and land rights revisited: Exploring new prospects via the state, family and market. *Journal of Agrarian Change*.

[b0015] Baada J.N., Baruah B., Luginaah I. (2019). What we were running from is what we’re facing again’: Examining the paradox of migration as a livelihood improvement strategy among migrant women farmers in the Brong-Ahafo Region of Ghana. *Migration and Development*.

[b0020] Baruah B. (2007). Assessment of public–private–NGO partnerships: Water and sanitation services in slums. *Natural Resources Forum*.

[b0025] Baruah, B. 2021. Women on Wheels in New Delhi, India: Can Social Innovation Promote Gender Equality? In *Social Economy in Asia: Realities and Perspectives,* edited by Euiyoung Kim and Hiroki Miura, 173-196. Lexington Books.

[b0030] Baruah B., Najjar D. (2022). *Gender Equality, Climate Change and Agriculture in the MENA region: Priorities and Possibilities*.

[b0035] Becerril J., Abdulai A. (2010). The impact of improved maize varieties on poverty in Mexico: A propensity score-matching approach. *World Development*.

[b0040] Beuchelt, Tina D. 2016. Gender, social equity and innovations in smallholder farming systems: Pitfalls and pathways. In *Technological and institutional innovations for marginalized smallholders in agricultural development,* edited by Franz W. Gatzweiler and Joachim von Braun, 181-198. Springer International Publishing.

[b0050] Bigler C., Amacker M., Ingabire C., Birachi E. (2017). Rwanda's gendered agricultural transformation: A mixed-method study on the rural labour market, wage gap and care penalty. *Women's Studies International Forum*.

[b0055] Brule R.E. (2020).

[b0060] Brush S.B., Corrales M.B., Schmidt E. (1988). Agricultural development and maize diversity in Mexico. *Human Ecology*.

[b0065] Buehren N., Goldstein M., Molina E., Vaillant J. (2017).

[b0070] CABI. 2020. Empowering female farmers: Gender responsive programming. 10.1079/CABICOMM-62-8121.

[b0075] Clawson D.L., Hoy D.R. (1979). Nealtican, Mexico: A peasant community that rejected the ‘Green Revolution’. *American Journal of Economics and Sociology*.

[b0080] Cleaver H.M. (1972). The contradictions of the Green Revolution. *The American Economic Review*.

[b0085] Das R.J. (1999). Geographical unevenness of India's green revolution. *Journal of Contemporary Asia*.

[b0090] Dewan R. (2016). Contextualising and visibilising gender and work in Rural India: Economic contribution of women in agriculture. *Indian Journal of Agricultural Economics*.

[b0095] Dhanagare D.N. (1987). Green Revolution and Social Inequalities in Rural India. *Economic and Political Weekly*.

[b0100] Doss C., Kovarik C., Peterman A., Quisumbing A., Van Den Bold M. (2015). Gender inequalities in ownership and control of land in Africa: Myth and reality. *Agricultural Economics*.

[b0105] Drucza K., Peveri V. (2018). Literature on gendered agriculture in Pakistan: Neglect of women's contributions. *Women's Studies International Forum*.

[b0110] Fafchamps M., Udry C., Czukas K. (1998). Drought and saving in West Africa: Are livestock a buffer stock?. *Journal of Development Economics*.

[b0115] Feldman S., Biggs S., Bruce Campbell W., Ortíz S.L. (2012). *Integrating agriculture, conservation and ecotourism: Societal influences*.

[b0120] Feldman S., Welsh R. (1995). Feminist knowledge claims, local knowledge, and gender divisions of agricultural labor: Constructing a successor science 1. *Rural Sociology*.

[b0125] Fisher M., Carr E.R. (2015). The influence of gendered roles and responsibilities on the adoption of technologies that mitigate drought risk: The case of drought-tolerant maize seed in eastern Uganda. *Global Environmental Change*.

[b0130] Frankel, F.R. (1971) 2015. *India’s Green Revolution: Economic Gains and Political Costs.* Princeton NJ: Princeton University Press.

[b0135] Freebairn D.K. (1995). Did the Green Revolution concentrate incomes? A quantitative study of research reports. *World Development*.

[b0140] Hänke H., Barkmann J. (2017). Insurance function of livestock, Farmers coping capacity with crop failure in southwestern Madagascar. *World Development*.

[b0145] Harris B., Jean Dreze J., Sen A. (1990). *The political economy of hunger: volume 1.entitlement and well-being*.

[b0150] Harris-White B., Haddad L., Hoddinot J., Harold Alderman H. (1997). *Intrahousehold resource allocation in developing countries: Models, methods and policy*.

[b0155] Harwood J. (2012).

[b0160] ICAR-IIWBR. 2017. *Progress Report of AICRP on Wheat and Barley 2016-17*. Karnal, Haryana: Indian Institute of Wheat and Barley Research (ICAR).

[b0165] ICRISAT. 2012. Dryland Cereals: A global alliance for improving food security, nutrition and economic growth for the world’s most vulnerable poor. Accessed September 14, 2022. https://gatesopenresearch.org/documents/3-1054.

[b0170] Kabeer, N. 2018. *Gender, livelihood capabilities and women’s economic empowerment: reviewing evidence over the life course*. Gender and Adolescence: Global Evidence (GAGE), London, UK.

[b0175] Kaviraj S. (1988). A critique of the passive revolution. *Economic and Political Weekly*.

[b0045] Kerr R.B. (2012). Lessons from the old Green Revolution for the new: Social, environmental and nutritional issues for agricultural change in Africa. Progress in Development Studies.

[b0180] Kilby P. (2019).

[b0185] Krishna V.V., Yigezu Y.A., Karimov A.A., Erenstein O. (2020). Assessing technological change in agri-food systems of the Global South: A review of adoption-impact studies in wheat. *Outlook on Agriculture*.

[b0190] Leach M. (2015). *Gender equality and sustainable development*.

[b0195] Lerner A.B. (2018). Political neo-Malthusianism and the progression of India’s green revolution. *Journal of Contemporary Asia*.

[b0200] Manfre C., Rubin D., Allen A., Summerfield G., Colverson K., Akeredolu M. (2013).

[b0205] Moseley W., Schnurr M., Kerr R.B. (2015). Interrogating the technocratic (neoliberal) agenda for agricultural development and hunger alleviation in Africa. *African Geographical Review*.

[b0210] Najjar D., Baruah B., El Garhi A. (2019). Women, irrigation and social norms in Egypt: ‘The more things change, the more they stay the same?’. *Water Policy*.

[b0215] Najjar D., Baruah B., El Garhi A. (2020). Gender and asset ownership in the old and new lands of Egypt. *Feminist Economics*.

[b0220] Najjar, D., Monika, P., Bipasha, B., Aden A. Aw-Hassan, and Libor, S. (2017). *Women, Decent Work and Empowerment in Rural Egypt*. Beirut, Lebanon: International Center for Agricultural Research in the Dry Areas (ICARDA). 10.13140/RG.2.2.30862.43848.

[b0225] Najjar D., Baruah B., Aw-Hassan A., Bentaibi A., Kassie G.T. (2018). Women, work, and wage equity in agricultural labour in Saiss, Morocco. *Development in Practice*.

[b0230] Negin J., Remans R., Karuti S., Fanzo J.C. (2009). Integrating a broader notion of food security and gender empowerment into the African Green Revolution. *Food Security*.

[b0235] Newton A.C., Flavell A.J., George T.S., Leat P., Mullholland B., Ramsay L., Revoredo-Giha C. (2011). Crops that feed the world 4. Barley: A resilient crop? Strengths and weaknesses in the context of food security. *Food Security*.

[b0240] Patel R. (2013). The long green revolution. *The Journal of Peasant Studies*.

[b0245] Patel R., Kerr R.B., Shumba L., Dakishoni L. (2015). Cook, eat, man, woman: Understanding the New Alliance for Food Security and Nutrition, nutritionism and its alternatives from Malawi. *Journal of Peasant Studies*.

[b0250] Pearse, A. (1980) 2015. Seeds of plenty, seeds of want: social and economic implications of the green revolution. In *Revisiting Sustainable Development*, 2015 (Reprinted), edited by Peter Utting, 139-157. Geneva: UNRISD.

[b0255] Perkins J.H. (1990). The Rockefeller Foundation and the green revolution, 1941–1956. *Agriculture and Human Values*.

[b0260] Pfitzer M., Krishnaswamy R. (2007).

[b0265] Pingali P.L. (2012). Green revolution: Impacts, limits, and the path ahead. *Proceedings of the National Academy of Sciences*.

[b0270] Pinstrup-Andersen P., Hazell P.BR. (1985). The impact of the Green Revolution and prospects for the future. *Food Reviews International*.

[b0275] Ragetlie R., Najjar D., Oueslati D. (2022). “Dear Brother Farmer”: Gender-Responsive Digital Extension in Tunisia during the COVID-19 Pandemic. *Sustainability*.

[b0280] SABMiller (2010). *Business Call to Action*.

[b0285] Saini S., Chowdhury A. (2023).

[b0290] Smale M., Bellon M.R., Gomez J.A.A. (2001). Maize diversity, variety attributes, and farmers’ choices in Southeastern Guanajuato, Mexico. *Economic Development and Cultural Change*.

[b0295] Singh, S. 2013. Sustainability & India Inc: SABMiller’s Saanjhi Unnati helps farmers earn higher incomes. *The Economic Times*, November 26. https://economictimes.indiatimes.com/industry/services/retail/sustainability-india-inc-sabmillers-saanjhi-unnati-helps-farmers-earn-higher-incomes/articleshow/26385999.cms.

[b0300] Sonnenfeld D.A. (1992). Mexico's “green revolution”, 1940-1980: Towards an environmental history. *Environmental History Review*.

[b0305] Times of India. 2009. Barley promises riches to farmers. 6 February. https://timesofindia.indiatimes.com/city/jaipur/Barley-promises-riches-to-farmers/articleshow/4083889.cms.

[b0310] Twyman J., Muriel J., García M.A. (2015). Identifying women farmers: Informal gender norms as institutional barriers to recognizing women’s contributions to agriculture. *Journal of Gender, Agriculture and Food Security*.

[b0315] Vercillo S., Kuuire V.Z., Armah F.A., Luginaah I. (2015). Does the new alliance for food security and nutrition impose biotechnology on smallholder farmers in Africa?. *Global bioethics*.

[b0320] Wellhausen E.J. (1976). The agriculture of Mexico. *Scientific American*.

